# Concha Bullosa of the Inferior Turbinate

**DOI:** 10.7759/cureus.19089

**Published:** 2021-10-28

**Authors:** Abdullah M Alnatheer, Feras Alkholaiwi

**Affiliations:** 1 College of Medicine, Imam Mohammad Ibn Saud Islamic University, Riyadh, SAU; 2 Otorhinolaryngology-Head and Neck Surgery, College of Medicine, Imam Mohammad Ibn Saud Islamic University, Riyadh, SAU

**Keywords:** nasal concha, turbinates, sinusitis, prognosis, radiography, systematic review

## Abstract

Inferior concha bullosa is a rare disease that has been related to sinonasal symptoms. This study aimed to determine the characteristics of concha bullosa in the inferior turbinate and assess its common clinical manifestations, computed tomography findings, and the surgical techniques used to treat the condition.

We conducted a search of the PubMed database, using the Preferred Reporting Items of Systematic Reviews and Meta-Analysis, for articles published until May 2021 using the following terms: “inferior turbinate and concha bullosa”; “inferior turbinate and pneumatization”; and in combination with other terms such as “concha bullosa release and concha bullosa excision”, among others.

We found 12 papers that met our inclusion criteria. The main presenting symptom was a nasal obstruction in 13 patients (100%), followed by headache in 10 patients (77%). The site of inferior concha bullosa was bilateral in six cases and left-sided in five cases. Computed tomography was conducted in all cases in this review. The type of intervention performed was medical, surgical, and both in 23%, 54%, and 23% of the cases.

Despite multimodal surgical approaches and medical treatment, all the outcomes were good and no compactions were noted. All cases also had good prognoses.

## Introduction and background

Sinonasal disease is one of the diseases frequently encountered in primary care and otorhinolaryngology clinics. A pneumatized turbinate is termed concha bullosa (CB), which refers to the presence of an air cell within a nasal turbinate [1]. It is one of the most common sinonasal anatomic variations. It is also a common variant of the nasal cavity among those with chronic sinusitis (49.5%) [2-3]. However, the pathophysiology underlying the pneumatization of the turbinate remains unclear [4].

The lateral nasal wall consists of three turbinates, namely, the superior, middle, and inferior turbinates, which have crucial roles in warming, humidifying, and filtering inspired air [5]. Concha bullosa occurs rarely in the inferior turbinate. It is more common in the middle turbinate, followed by the superior turbinate [1]. A computed tomography (CT) scan remains the best diagnostic modality for CB. A radiological study assessed 594 CT scans that revealed the incidence of middle turbinate CB at 53.7% compared to only 1% (6/594) for the inferior turbinate CB, which were mostly unilateral cases [4]. Moreover, another study reported that most of the pneumatization was unilateral in 14 (88%) patients and bilateral in two (12%) patients [1].

Pneumatized inferior turbinates (PITs) may be symptomatic or may be detected coincidentally, particularly during a CT scan [4,6]. The main clinical manifestations include nasal obstruction and headache, especially when the pneumatization is extensive; this should be evaluated via nasal endoscopy [7]. Additionally, there is a correlation between CB and septal deviation (SD), where SD may result from a predominantly ipsilateral CB [8-9].

There are only a few studies that have reported cases of pneumatization of the inferior turbinate. In this review, we aimed to determine the characteristics of CB in the inferior turbinate, its clinical manifestations, other CT findings, and management strategies, including surgical techniques.

## Review

Methods

**Figure 1:**
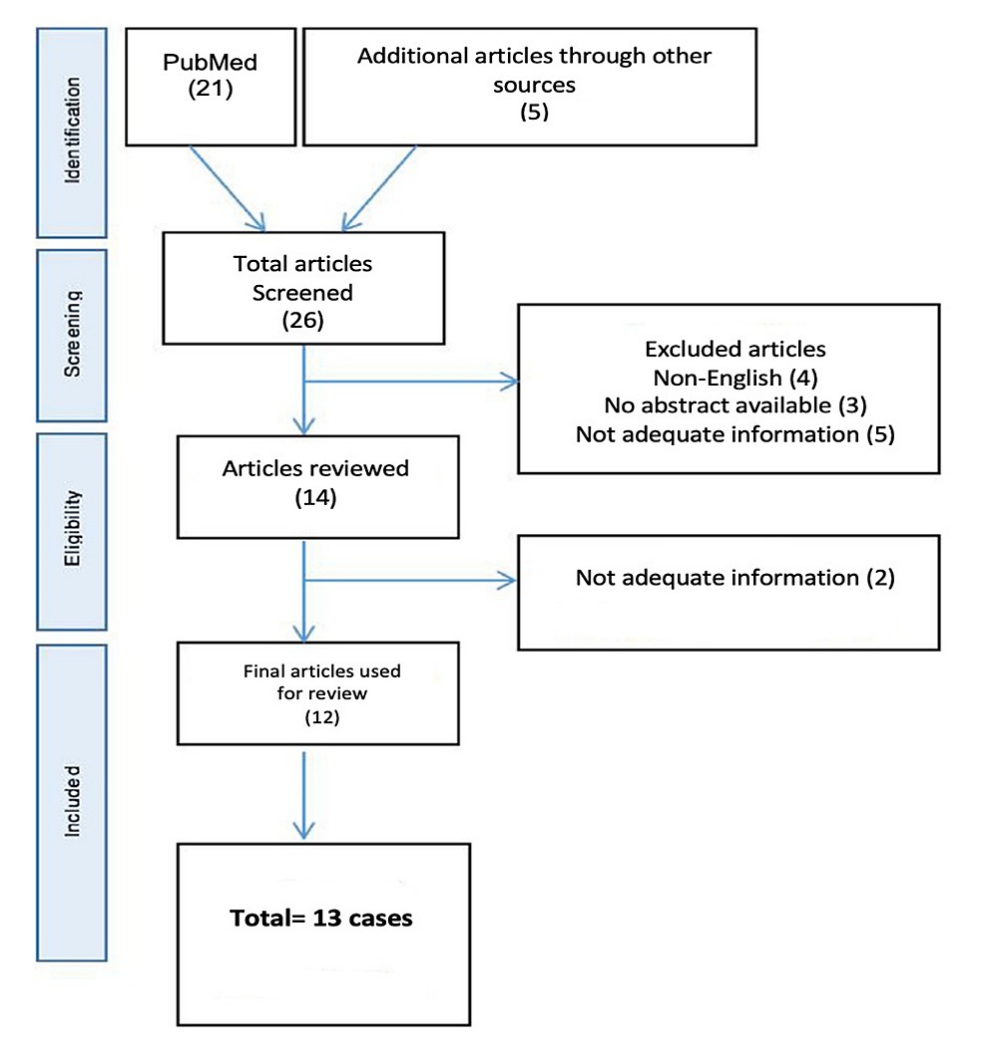
PRISMA flow diagram PRISMA: Preferred Reporting Items of Systematic Reviews and Meta-Analysis

A literature review was conducted to find all published cases of CB (pneumatization) in the inferior turbinate, using the Preferred Reporting Items of Systematic Reviews and Meta-Analysis [10], by exploring the PubMed database (Figure 1). Two investigators (A.M.N. and F.M.K.) independently examined all articles in a standardized manner to determine eligibility and, subsequently, compare highlighted articles. The search was conducted in May 2021 using the following keywords: ((“inferior turbinate”) AND (“concha bullosa”)); ((“inferior turbinate”) AND (“pneumatization”)) alone; and in combination with other words such as (“concha bullosa release”), (“concha bullosa excision”), (“endoscopic sinus surgery”), (“antrostomy”), and (“septoplasty”) to decrease the possibility of missed cases.

Our search included all articles that have reported the prevalence of CB in the inferior turbinate between 1989 and 2021. Data were collected on age, gender, presenting symptoms, physical examination findings, associated findings in the CT scan, type of intervention, surgical technique used, and the presence of complications related to the procedure. The articles that have reported CB in the middle or superior turbinate alone were excluded. Only literature in the English language was included in our research.

Results

The literature search identified 26 papers, which were all case reports that have reported pneumatization of the inferior turbinate between 1999 and 2021. Seven articles were excluded because they were missing significant information regarding inclusion criteria, and five were excluded either because they were non-English language publications or they lacked an abstract. We included 12 case reports in this review, wherein a total of 13 patients were diagnosed with inferior turbinate CB through history assessment, physical examination, and imaging (Table 1). The mean age of the patients was 27 years old, and the majority were females, comprising 69.2% of the cases while 30.8% were males (Table 2).

**Table 1:** Summary of the articles that report concha bullosa in the inferior turbinate CB: concha bullosa; DNS: deviated nasal septum

Reference	Number of Patients	Age	Gender	Site	Associated findings in CT scan
Pittore et al [11].	1	27	Female	Left	Polypoid mucosal thickening bilaterally in the maxillary antrum and ethmoid air cell, left infundibulum occluded, inferior turbinates hypertrophic and concha bullosa in the left side
San T et al [12].	1	20	Female	Bilateral	Inferior, middle, and superior turbinates were pneumatised bilaterally, a moderate nasal septum deviation to the left with a spur formation and inflammatory mucosal thickening in the right maxillary sinus
Göçmen et al [13].	1	52	Male	Right	Bilateral inferior and left middle concha bullosa, left over-pneumatized ethmoid bulla, posterior septal pneumatization, and right septal deviation
Koo et al [14].	1	14	Male	Bilateral	Bilateral pneumatization of the inferior turbinate and hypertrophy of the left inferior turbinate, two concha bullosa were located at the posterosuperior and posteroinferior portions of the left inferior turbinate
Kiroglu et al [15].	1	14	Female	Left	Pneumatization of the left inferior turbinate
Ozcan et al [16].	1	35	Female	Bilateral	Bilateral inferior and left middle concha bullosa, left over-pneumatized ethmoid bulla, posterior septal pneumatization, and right septal deviation
Toplu et al [17].	1	37	Female	Bilateral	Bilateral inferior CBs, which were directly communicated with ipsilateral maxillary sinuses, septated right middle CB, bilateral superior CB’s, right uncinate pneumatization, septal deviation to the left side, and sinusitis
Fidan V [18].	1	17	Female	Bilateral	Concha bullosa in all turbinates with almost total nasal obstruction
Giourgos et al [19].	1	11	Female	Left	Left inferior concha bullosa and bilateral pneumatization of the uncinate process and the Haller cells
Aydın et al [20].	1	35	Female	Bilateral	Pneumatization of the inferior turbinate bilaterally analogous to a lamellar type pneumatization of the middle turbinate., septal deviation to left
Dogru et al [21].	1	35	Female	Right	Right middle and inferior turbinate pneumatizations
Alkhaldi et al [22].	2	32	Male	Left	DNS to the right, bilateral CB of the middle turbinate, paradoxical right middle turbinate, and CB of the left inferior turbinate
		28	Male	Left	Bilateral CB of the middle turbinate, predominantly on the left side, CB of the left inferior turbinate with bilateral inferior turbinate hypertrophy

**Table 2:** Patient demographics.

Number of cases	% of males	% of females	Age range (years)	Mean age (years)	Median age (years)
13	30.8%	69.2%	11-52	27	28

**Figure 2:**
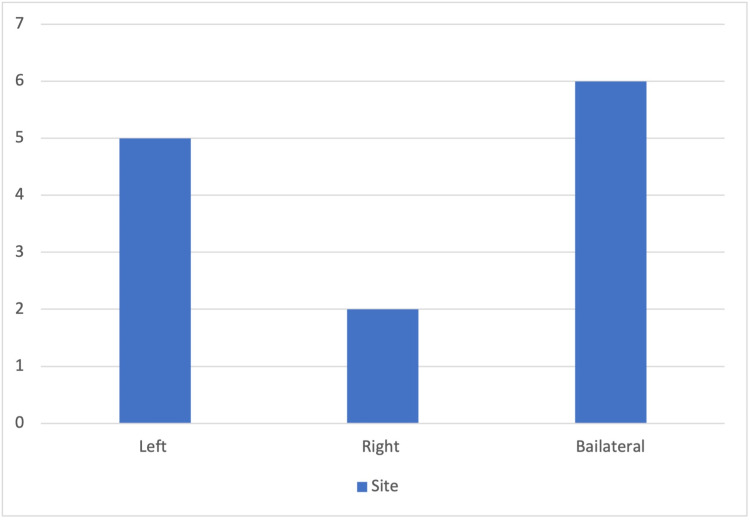
Site of concha bullosa in the inferior turbinate

All 13 patients (100%) presented with nasal obstruction, followed by headache in 77% (10/13) of the patients and septal deviation in 61% (8/13) of the cases (Table 3). The site of CB in the inferior turbinate was bilateral in six cases, followed by left-sided in five cases and right-sided in two cases (Figure 2). Inferior, middle, and superior turbinate pneumatization was reported in two cases, and six were associated with the middle turbinate.

**Table 3:** Common presenting symptoms physical examination findings

Clinical manifestations	N (%)
Symptoms:	
Nasal obstruction	13 (100%)
Headache	10 (77%)
Postnasal drip	7 (53%)
Purulent nasal discharge	3 (23%)
Physical examination findings:	
Inferior turbinate hypertrophy	12 (92%)
Middle turbinate hypertrophy	4 (30%)
Septal deviation	8 (61%)

Our results revealed that the type of intervention performed was solely medical in three patients, surgical in seven, and both medical and surgical interventions in three. All patients exhibited improvements, and no surgical complications were reported (Table 4).

**Table 4:** Intervention and outcome

Reference	Number of Patients	Intervention (medical / surgical)	Surgical technique	Outcome
Pittore et al [11].	1	Surgical	The inferior concha bullosa (ICB) was resected removing the free edge of the inferior turbinate using turbinectomy scissors) was performed bilaterally	No complications, the patient improved
San et al [12].	1	Surgical	Crushing and outfracture of the inferior turbinates. For both inferior turbinate pneumatizations, the bullous structures were reduced by crushing and outfracture.	No complications, the patient improved
Göçmen et al [13].	1	Surgical	The infected and pneumatized inferior turbinate was opened and purulent material drained; the lateral lamella of the turbinate was resected, preserving the mucosa of the inferior turbinate.	No complications, the patient improved
Koo et al [14].	1	Surgical	Endoscopic left middle meatal antrostomy, left inferior turbinectomy, and lateral out-fracture of both inferior turbinates.	No complications, the patient improved
Kiroglu et al [15].	1	Both	Under local anesthesia, the lateral lamella of the inferior concha bullosa was resected.	No complications, the patient improved
OZCAN et al [16].	1	Surgical	Bilateral inferior concha outfracture	No complications, the patient improved
Toplu et al [17].	1	Both	Lateral lamella of the right middle concha was resected, superior CB was crushed and outfracture, crushing, and radiofrequency thermocoagulation were performed to inferior turbinate.	No complications, the patient improved
Choi et al [18].	1	Both	The middle and inferior concha bullosae were resected, removing the free edge of the inferior turbinate using turbinectomy scissors.	No complications, the patient improved
Giourgos et al [19].	1	Medical	-	The patient improved
Aydın et al [20]	1	Surgical	Septoplasty / no turbinate surgery done	No complications, the patient improved
Dogru et al [21].	1	Surgical	Underwent endoscopic sinus surgery under local topical anesthesia. Using a 0”, 4-mm endoscope and sickle knife, an incision was performed in the anterior aspect of the inferior turbinate. Once the pneumatized air cell was entered, the incision was extended superiorly and inferiorly with the appropriately angled sinus scissors. The lateral portion of the turbinate was excised in an anterior to posterior direction.	No complications, the patient improved
Alkhaldi et al [22].	2	Medical	Male	Patients improved

Discussion

Concha bullosa in the inferior turbinate is a rare condition, with limited cases reported in the existing literature. A retrospective study conducted in Japan between 2000 and 2004 on 2,500 cases with coronal paranasal sinus CT has revealed 10 patients with PITs, with an incidence of one in 250 patients (6 females and 4 males) and with an average age of 29.7 years old (range, 7-60 years old). These findings are consistent with the findings of this review, suggesting that PIT is possibly linked to articulation defects between the maxillary process of the palatine bone and the maxillary bone [23]. Moreover, another study that reviewed CT scans for a period of 12 years discovered 16 cases of PIT (0.03%) [1]. A study has reported nasal obstruction (56%) and headache (25%) as the most frequent complaints. In comparison to our findings, all patients in that study experienced nasal obstruction (100%), and 77% experienced headache or facial pain, which was due to the mucosal contact point [1,7]. We observed an association between SD and CB in the inferior turbinates, where 61% had SD on endoscopic evaluation. Diagnostic imaging, especially CT, is crucial to evaluate and manage PITs. In our review, all cases underwent CT before treatment interventions were performed.

Patients with asymptomatic CB usually require no medical or surgical interventions. In contrast, if the patient is symptomatic, the management strategies could vary from medical to surgical interventions. However, for middle turbinate CB, which has a higher prevalence in comparison to inferior concha bullosa (ICB), several surgical interventions have been published in the literature such as crushing with intrinsic stripping techniques in CB surgery. This technique showed a significant reduction in the CB size upon measuring the presurgical and one-year postsurgical endoscopic nasal cavity images and CT scans of all the patients who were included in the study [24]. Another study has shown a significantly decreased postoperative CB size by making a horizontal incision with a no. 12 blade along the inferior aspect of the CB from a posterior to anterior direction. A vertical incision was then made with a no. 12 blade along the anterior aspect of the CB, meeting the horizontal incision to form an “L.” Both incisions were infiltrated through the mucosa and underlying bone. The medial wall of the CB was then lateralized using a blunt instrument, with no postoperative complications [25].

In 1988, Zinreich et al. were the first to observe PITs as an anatomical variant of the turbinates [26]. Multiple surgical techniques have been characterized, such as outfracturing of the inferior turbinate and crushing of the ICB with forceps, excision of the free edge of the inferior turbinate using turbinectomy scissors, submucosal diathermy, and turbinoplasty with the use of a microdebrider [27-29]. If the pneumatization occurs anteriorly without any posterior involvement, the CB can be treated by partial turbinectomy, removing only the anterior portion to protect as much of the turbinate as possible. Curved scissors should be placed at the neck of the turbinate just above the bulge of the area of the pneumatization, with the curve pointing inferiorly. In the event of an extremely large pneumatization, a lateral turbinectomy may be used as described by Dogru et al. in 1999, wherein the ICB is resected through the removal of the lateral aspect of the inferior turbinate. Since then, several other authors have used this method [15,21,30]. Although this technique is not difficult, it is contraindicated when there is an association between the ICB and the maxillary sinus due to the possibility of producing an inferior meatal antrostomy, which may cause mucociliary recirculatory issues [21].

Unlu et al. have recommended the use of a sickle knife to perform a vertical incision along the anteroinferior surface of the turbinate, followed by the use of a Blakesley-Wilde forceps to remove the inferior mucosa from the ICB [28]. However, crushing may be sufficient to relieve the nasal obstruction for a small CB. In this technique, the turbinate is grasped with pituitary forceps or using a Freer dissector inserted between the septum and the turbinate and directed laterally to crush the turbinate against the lateral wall or the Freer dissector inserted lateral to the CB to crush it against the septum [31].

A 4-mm 0-degree rigid endoscope was used in a case report, and the ICB was resected by removing the free edge of the inferior turbinate using turbinectomy scissors [11]. Moreover, a total turbinectomy is associated with an increased risk of developing atrophic rhinitis; thus, it is contraindicated [32].

## Conclusions

In conclusion, inferior CB is a rare disease associated with sinonasal symptoms. Most patients presented with nasal obstruction and headache. Despite multimodal surgical approaches and medical treatment, all the outcomes were good and showed no compactions, with a good prognosis. We recommend further studies to understand the pathophysiology of the disease and raise the high index of suspicion for our differential diagnosis.

## References

[1] Yang BT, Chong VF, Wang ZC, Xian JF, Chen QH (2008). CT appearance of pneumatized inferior turbinate. Clin Radiol.

[2] Belli E, Rendine G, Mazzone N (2009). Concha bullosa. Endoscopic treatment. J Craniofac Surg.

[3] Subramanian S, Lekhraj Rampal GR, Wong EF, Mastura S, Razi A (2005). Concha bullosa in chronic sinusitis. Med J Malaysia.

[4] Koo SK, Kim JD, Moon JS, Jung SH, Lee SH (2017). The incidence of concha bullosa, unusual anatomic variation and its relationship to nasal septal deviation: a retrospective radiologic study. Auris Nasus Larynx.

[5] Baraniuk J, Kim D (2007). Nasonasal reflexes, the nasal cycle, and sneeze. Curr Allergy Asthma Rep.

[6] Tiwari R, Goyal R (2019). Role of concha bullosa in chronic rhinosinusitis. Indian J Otolaryngol Head Neck Surg.

[7] Kantarci M, Karasen RM, Alper F, Onbas O, Okur A, Karaman A (2004). Remarkable anatomic variations in paranasal sinus region and their clinical importance. Eur J Radiol.

[8] Sazgar AA, Massah J, Sadeghi M, Bagheri A, Rasool E (2008). The incidence of concha bullosa and the correlation with nasal septal deviation. B-ENT.

[9] Yiğit O, Acioğlu E, Cakir ZA, Sişman AS, Barut AY (2010). Concha bullosa and septal deviation. Eur Arch Otorhinolaryngol.

[10] Moher D, Liberati A, Tetzlaff J, Altman DG (2009). Preferred reporting items for systematic reviews and meta-analyses: the PRISMA statement. PLoS Med.

[11] Pittore B, Al Safi W, Jarvis SJ (2011). Concha bullosa of the inferior turbinate: an unusual cause of nasal obstruction. Acta Otorhinolaryngol Ital.

[12] San T, San S, Gürkan E, Erdoğan B (2014). Bilateral triple concha bullosa: a very rare anatomical variation of intranasal turbinates. Case Rep Otolaryngol.

[13] Göçmen H, Oğuz H, Ceylan K, Samim E (2005). Infected inferior turbinate pneumatization. Eur Arch Otorhinolaryngol.

[14] Koo SK, Moon JS, Jung SH, Mun MJ (2018). A case of bilateral inferior concha bullosa connecting to maxillary sinus. Braz J Otorhinolaryngol.

[15] Kiroglu AF, Cankaya H, Yuca K, Kara T, Kiris M (2007). Isolated turbinitis and pneumatization of the concha inferior in a child. Am J Otolaryngol.

[16] Ozcan C, Görür K, Duce MN (2002). Massive bilateral inferior concha bullosa. Ann Otol Rhinol Laryngol.

[17] Toplu Y, Bayindir T, Karatas E, Akarcay M (2013). All concha bullosa: an undefined abnormality of the lateral nasal wall. Indian J Otolaryngol Head Neck Surg.

[18] Fidan V (2012). Panconcha bullosa. New definition in the literature. J Craniofac Surg.

[19] Giourgos G, Matti E, Carena P, Pagella F (2010). A unique case of multiple sites of pneumatization of the sinonasal bony framework in a pediatric patient. Ear Nose Throat J.

[20] Aydın Ö, Üstündağ E, Çiftçi E, Keskin I (2001). Pneumatization of the inferior turbinate. Auris Nasus Larynx.

[21] Doǧru H, Döner F, Uygur K, Gedikli O, Çetin M (1999). Pneumatized inferior turbinate. Am J Otolaryngol.

[22] Alkhaldi AS, Alhedaithy R, Alghonaim Y (2021). Concha bullosa of the inferior turbinate: report of two cases. Cureus.

[23] Oztürk A, Alataş N, Oztürk E, San I, Sirmatel O, Kat N (2005). Pneumatization of the inferior turbinates. Incidence and radiologic appearance. J Comput Assist Tomogr.

[24] Eren SB, Kocak I, Dogan R, Ozturan O, Yildirim YS, Tugrul S (2014). A comparison of the long-term results of crushing and crushing with intrinsic stripping techniques in concha bullosa surgery. Int Forum Allergy Rhinol.

[25] Mesbahi A, Movahhedian N, Akbarizadeh F, Hakimi AA, Khojastepour L (2021). Assessing the efficacy of a modified crushing technique for the management of concha bullosa: a cone beam computer tomography study. Braz J Otorhinolaryngol.

[26] Zinreich SJ, Mattox DE, Kennedy DW, Chisholm HL, Diffley DM, Rosenbaum AE (1988). Concha bullosa: CT evaluation. J Comput Assist Tomogr.

[27] Cankaya H, Egeli E, Kutluhan A, Kiriş M (2001). Pneumatization of the concha inferior as a cause of nasal obstruction. Rhinology.

[28] Unlu HH, Altuntas A, Aslan A, Eskiizmir G, Yucel A (2002). Inferior concha bullosa. J Otolaryngol.

[29] Ozcan KM, Gedikli Y, Ozcan I, Pasaoglu L, Dere H (2008). Microdebrider for reduction of inferior turbinate: evaluation of effectiveness by computed tomography. J Otolaryngol Head Neck Surg.

[30] Uzun L, Ugur MB, Savranlar A (2004). Pneumatization of the inferior turbinate. Eur J Radiol.

[31] Cannon CR (1994). Endoscopic management of concha bullosa. Otolaryngol Head Neck Surg.

[32] Clement WA, White PS (2001). Trends in turbinate surgery literature: a 35-year review. Clin Otolaryngol Allied Sci.

